# Different Tokes for Different Folks: Use of Cannabis Products Among a Longitudinal Cohort of People with Heroin Dependence

**DOI:** 10.1007/s11469-023-01071-5

**Published:** 2023-05-15

**Authors:** Jack Wilson, Katherine L. Mills, Matthew Sunderland, Tom P. Freeman, Maree Teesson, Paul S. Haber, Christina Marel

**Affiliations:** 1 The Matilda Centre for Research in Mental Health and Substance Use, Level 6, Jane Foss Russell Building, G02, The University of Sydney, Sydney, NSW 2006 Australia; 2 Addiction and Mental Health Group (AIM), University of Bath, Bath, BA2 7AY UK; 3 Sydney Medical School, The University of Sydney, Sydney, NSW 2006 Australia; 4 Drug Health Services, Royal Prince Alfred Hospital, Camperdown, NSW 2050 Australia

**Keywords:** Cannabis, Heroin, Opioid use disorder, Tetrahydrocannabinol, Longitudinal, Cohort

## Abstract

Co-occurring cannabis use is common among those with opioid use disorder (OUD), but the extent to which it is harmful may be due to its preparation and concentration of various cannabinoids. The current study aimed to examine the prevalence of, and long-term associations with, the use of varying cannabis products among a naturalistic longitudinal cohort of people with heroin dependence. A total of 615 people, most of whom were entering treatment, were recruited to the Australian Treatment Outcome Study (ATOS) in 2001–2002. This analysis focuses on the 401 participants followed up at 18–20 years post baseline. Structured interviews assessed the use of cannabis products, as well as demographic and health covariates. High-potency/indoor-grown cannabis was the most common type ever used (68.8%), and in the past 12 months (80.4%), followed by low potency/outdoor grown (22.4%; 14.4%), and less so for other types of cannabis. After controlling for covariates, older age at baseline was associated with lower odds of high-potency cannabis being used as the primary type in the past 12 months. In contrast to studies of non-opioid dependent populations, common use of high-potency cannabis was not associated with more severe health outcomes.

**Supplementary Information:**

The online version contains supplementary material available at 10.1007/s11469-023-01071-5.

## Introduction

Polydrug use is common among those with opioid use disorders (OUD), which has generally been shown to exacerbate the existing ill health of this population (Compton et al., 2021). Around one to two-thirds of those with OUD report frequent cannabis use (Kral et al., 2015; Rosic et al., 2021), but this could become more prevalent as there is a growing interest in cannabis as a substitute or complement to opioids, either as an alternative analgesic or method of reducing withdrawal symptoms (Humphreys & Saitz, 2019; Wiese & Wilson-Poe, 2018). The extent to which cannabis is harmful or therapeutic is largely due to its preparation and concentration of various cannabinoids (T. P. Freeman et al., 2019a, 2019b). Given that those with OUD have an increased likelihood of experiencing adverse health outcomes (Degenhardt et al., 2018), it is imperative to understand the types of cannabis being consumed by this population, and the degree to which they may be exposed to further harm.

### Variation in Cannabis Products

Cannabis contains over 140 different exogenous cannabinoids of which delta-9-tetrahydrocannabinol (THC) and cannabidiol (CBD) are typically the most abundant (Hanuš et al., 2016). Concentrations of THC and CBD within cannabis largely depend on plant genetics, growing conditions, and method of preparation. For instance, unpollinated female plants (sinsemilla, hydro or skunk) can produce higher THC concentrations because they can divert energy from seed production to the synthesis of THC (Potter, 2013), whereas fertilised seeded plants (bush/outdoor grown) are typically less potent, often with around half the THC concentration but similar in CBD content (approx. < 1%). More recently, cannabis concentrates have emerged within the cannabis market, produced through a range of extraction techniques such as butane or super-critical carbon dioxide, containing extremely high concentrations of THC with negligible amounts of CBD (Raber et al., 2015). Despite variation in THC and CBD content between types of products, there has been an international trend towards the use of high-potency products (Freeman et al., 2021).

### The Effects of THC and CBD

THC is a partial agonist at cannabinoid type 1 receptors, resulting in psychoactive effects that are responsible for the ‘high’ that consumers experience (Bhattacharyya et al., 2010). Clinical trials demonstrate dose-dependent effects of THC on intoxication, cognitive impairment, anxiety, and psychotic-like symptoms (Curran et al., 2002; D'Souza et al., 2004). Whereas epidemiological studies suggest that the use of higher THC products is associated with a greater risk of developing cannabis use disorder (CUD) (Craft et al., 2019; Freeman & Winstock, 2015), psychotic conditions (Di Forti et al., 2019; Mackie et al., 2021), and some evidence of anxiety and depression (Chan et al., 2017; Morgan et al., 2012). Although those using cannabis have been shown to partially titrate their consumption with varying concentrations of THC (e.g. adjusting inhalation volume), the use of higher THC concentration products typically results in greater THC doses (Freeman et al., 2014; Leung et al., 2021).

While THC is associated with the harmful effects of cannabis, CBD has been considered as an alternative therapy for a range of health conditions (e.g. psychosis, epilepsy) (Bonaccorso et al., 2019). This non-psychoactive cannabinoid has a low affinity for cannabinoid receptors but is thought to exert its mechanism of action by altering the reuptake and efficacy of other cannabinoid agonists and antagonists (Campos et al., 2012; McPartland et al., 2015). Of relevance, recent clinical trials have demonstrated that CBD may be an effective treatment for both heroin use disorder (Hurd et al., 2019) and CUD (Freeman et al., 2020). However, the CBD doses administered in these studies far exceed concentrations observed in the cannabis market (Hurd et al., 2019).

When consumed together, CBD may attenuate some of the negative effects of THC (A. M. Freeman et al., 2019a, 2019b). Within naturalistic studies, those using cannabis with greater CBD content were less likely to exhibit cognitive impairments, psychotic-like experiences, and anxiety disorders (Demirakca et al., 2011; Morgan & Curran, 2008; Morgan et al., 2010a, 2010b; Morgan et al., 2010a, 2010b). However, clinical evidence that CBD protects against the acute harms of THC is not as clear (A. Englund et al., 2013; A. Englund et al., 2022). In terms of polydrug use, a UK longitudinal birth cohort followed up at 24 years of age found that those most commonly using high-potency cannabis were more than twice as likely to report recent use of other drugs, and three times more likely to report tobacco dependence compared to those using low potency cannabis (Hines et al., 2020). However, these associations were no longer significant after adjusting for sociodemographic factors, age of cannabis onset, and frequency of cannabis use.

Despite high rates of co-occurring cannabis use among those with other substance use disorders (Hasin & Walsh, 2020), studies have yet to assess the prevalence and impact of various cannabis products on this population. Considering that those with OUD experience high rates of psychiatric and physical comorbidities, co-consumption of cannabis may have varying effects according to the type of cannabis product used. As seen in both clinical and naturalistic studies, it may therefore be expected that those using cannabis products with higher THC and lower CBD would experience more severe physical and psychiatric conditions.

### Current Study

The current study aims to assess the prevalence of, and long-term associations with, the use of varying cannabis products among a naturalistic longitudinal cohort of people with heroin dependence. This will be achieved by (a) assessing the prevalence, frequency, and quantity of past 12-month use of varying cannabis products and the most common type of cannabis product used over a person’s lifetime, (b) examining the relationship between baseline covariates and the use of varying cannabis products, and (c) the relationship between the use of varying cannabis products and 18–20-year outcomes.

## Material and Methods

This study has been reported in accordance with the Strengthening the Reporting of Observational Studies in Epidemiology (STROBE) checklist (Supplementary table 1). The primary research question has not been pre-registered on a publicly available platform and therefore should be considered exploratory.

### Design

The Australian Treatment Outcome Study (ATOS) is a longitudinal prospective cohort study of people entering treatment for heroin dependence in Sydney, Australia (Ross et al., 2002). Consisting of 615 participants, 535 (87%) were entering maintenance therapies (*n* = 201), detoxification (*n* = 201), and residential rehabilitation (*n* = 133), while 80 were included as a non-treatment seeking sample, recruited from needle and syringe programs located within the same regional areas as treatment entrants. Eligibility criteria for participation included the following: (i) had no treatment for heroin dependence in the preceding month, (ii) were not a prisoner within a correctional facility in the preceding month, (iii) were aged 18 years or over, (iv) agreed to provide contact details for follow-ups, and (v) were proficient in English. Ethics approval for the 18–20-year follow-up was obtained from the Sydney Local Health District Royal Prince Alfred Zone (X18-0512 & HREC/18/RPAH/733).

Participants were recruited and interviewed at baseline in 2001–2002, before being followed up at 3 months, 12 months, 24 months, 36 months, 10–11 years, and 18–20 years; however, the current study will be limited to data at baseline and 18–20 years. At 18–20 years, 401 (65.2%) of the original sample were followed up, and the sample was largely similar in baseline characteristics compared to those not followed up, aside from age, where older age predicted drop-out (Marel et al., 2023). While all baseline interviews were conducted face to face, 71.6% of 18–20-year follow-up interviews were conducted via telephone, largely due to COVID-19 restrictions. Written informed consent were provided for both participation and to be contacted for future follow-ups. Participants were reimbursed $20 AUD at baseline and $50 AUD at 18–20 years.

### Measures

At 18–20 years, participants were assessed for the use of different cannabis products. Consistent with availability within Australian (Peacock et al., 2019) and international cannabis markets (ElSohly et al., 2016; Potter et al., 2018), participants were asked about their use (i.e. frequency, quantity on a typical occasion, most common type) of the following types of cannabis: high-potency herbal/indoor grown/sinsemilla, hash or resin, low-potency herbal/outdoor grown, and cannabis oil or concentrates. Prior to administering the cannabis-related questions, the interviewer provided a verbal description of each cannabis product to aid the participants’ recollection. Previous data have demonstrated strong validity between self-reported cannabis type and concentrations of THC (Freeman et al., 2014).

Baseline covariates included age, sex, county of birth, main source of income, and usual form of accommodation in the last month. The Opiate Treatment Index (OTI) was used to assess the past month substance use (i.e. cannabis, heroin, prescribed or non-prescribed opiates, alcohol, amphetamines, cocaine, benzodiazepines, antidepressants, hallucinogens, inhalants), injection-related risk-taking behaviours, as well as lifetime prison history and past month criminal involvement (Darke et al., 1992). General physical and mental health were measured using the 12-Item Short Form Health Survey (SF-12) which is composed of a Physical Component Summary (PCS) score and a Mental Component Summary (MCS) score (Ware et al., 1996). With an overall standardised mean score of 50 and standard deviation of 10 in the US population, higher summary scores indicate better health, and lower scores suggest poorer health. The Composite International Diagnostic Interview (CIDI) version 2.1 was used to assess a past month diagnosis of DSM-IV major depression, lifetime diagnosis of DSM-IV post-traumatic stress disorder (PTSD) (World Health Organisation, 1997), and lifetime suicidal ideation and attempt, while a modified version of the Diagnostic Interview Schedule (DIS) was used to screen for a DSM-IV diagnosis of antisocial personality disorder (ASPD). Baseline ICD-10 borderline personality disorder (BPD) was assessed using the International Personality Disorder Examination Questionnaire (IPDEQ) (Loranger et al., 1997). As per previous work by Darke et al. (1996), participants were also asked about lifetime and recent history of heroin overdose.

At 18–20 years, participants were readministered similar questions, with the addition of past month use of synthetics or new and emerging psychoactive substances (NPS), lyrica, and gabapentin. The CIDI version 2.1 was used to obtain a diagnosis for DSM-IV past month cannabis and heroin dependence at the 18–20-year follow-up (World Health Organisation, 1997). Those interviewed face to face were administered the Mini-Addenbrooke’s Cognitive Examination (Mini-ACE) Australian version, which assesses attention, memory, letter fluency, clock drawing, and memory recall (Hsieh et al., 2015). The assessment is scored out of 30, with a higher score indicating better cognitive functioning.

### Statistical Analysis

Prevalence of the most common cannabis type used over a person’s lifetime and in the past 12 months was calculated. To assess the prevalence of cannabis types used in the past 12 months, percentages were calculated for high potency/indoor grown, low potency/outdoor grown, hash/resin, and cannabis oil/concentrates. Further prevalence rates were conducted for frequency and quantity of each cannabis product used in the past 12 months. Due to low cell counts, frequency of use of different cannabis products in the past 12 months was categorised as ‘less than monthly’, ‘at least monthly’, and ‘daily’, while the quantity of use was categorised as ≤ 1, 2–5, and 5 > joints/cones on a typical occasion.

To examine the association between baseline variables and the use of varying cannabis products, binary logistic regressions were conducted with the most common type of cannabis used in the past 12 months as the dependent variable. Due to low cell counts, cannabis type was categorised as high-potency cannabis (high potency/indoor grown, hash/resin and cannabis oil/concentrates) with the comparator as low potency cannabis (low potency/outdoor grown, don’t know, and other). Univariable regressions were initially conducted to determine variables that were significantly associated with the outcome at *p*-value < 0.05, before being entered into a multivariable model. An identical analysis was carried out to examine the associations between 18–20-year outcomes and the most common type of cannabis in the past 12 months, while controlling for baseline covariates determined to be significant at univariable analysis. According to established interpretations of Variance Inflated Factor (VIF; acceptable levels < 10), independent variables within the multivariable models were not highly correlated (O’Brien, 2007). Statistical significance for the multivariable models was adjusted for multiple comparisons using the Bonferroni correction. All analysis was conducted using IBM SPSS Statistic 25.0.

## Results

### Prevalence of Cannabis Types Used at 18–20 years

At 18–20 years post-baseline, 397 (99.0%) of the 401 participants followed-up had ever used cannabis, and 209 (52.1%) had used in the past 12 months. As seen in Table 1, high potency/indoor grown (68.8%) was the most common type of cannabis ever used, followed by low potency/outdoor grown (22.4%), other (2.3%), and hash/resin (2.0%). Of those who reported ‘other’, six participants specified ‘50% high potency and 50% low potency’, one ‘50% hash and 50% low potency’, and two ‘buddha sticks or Thai sticks’. A small proportion (4.5%) did not know what kind of cannabis they had used most often, and there were no reports of cannabis oil/concentrates as the most common type of cannabis ever used.

**Table 1:** Prevalence of the most common type of cannabis ever used, and used in the past 12 months

	Cannabis type					
	High potency/indoor grown	Low potency/outdoor grown	Hash/resin	Cannabis oil/concentrates	Don’t know	Other
Most common type ever used (*n* = 397)	273 (68.8%)	89 (22.4%)	8 (2.0%)	0 (0.0%)	18 (4.5%)	9^a^ (2.3%)
Most common type used in the past 12 months (*n* = 209)	168 (80.4%)	30 (14.4%)	3 (1.4%)	1 (0.5%)	3 (1.4%)	4^b^ (1.9%)

^a^‘50% high potency and 50% low potency’—6, ‘50% hash and 50% low potency’—1, and ‘buddha sticks or Thai sticks’—2

^b^‘50% high potency and 50% low potency’—3, ‘edibles’—1

In terms of the most common type of cannabis used in the past 12 months, a majority reported high potency/indoor grown (80.4%), followed by low potency/outdoor grown (14.4%), hash/resin (1.4%), other (1.9%), and cannabis oils/concentrates (0.05%) (Table 1). Of those who responded ‘other’, three specified ‘50% high potency and 50% low potency’, and one ‘edibles’. Meanwhile, 1.4% did not know the most common type of cannabis used in the past 12 months.

Of those who had used cannabis in the past 12 months, 190 (90.9%) reported using high potency/indoor grown, 108 (51.7%) used low potency/outdoor grown, 21 (10.0%) used hash/resin, and 17 (8.1%) used cannabis oil/concentrates (Table 2). Among those who had used a cannabis product less than monthly, there was a similar proportion of participants using high potency/indoor grown (41.6%) to low-potency/outdoor-grown cannabis (40.9%). However, daily cannabis use typically consisted of high-potency/indoor-grown cannabis (82.9%). Hash/resin and cannabis oil/concentrates were infrequently reported and were predominantly used less than monthly (8.7%; 8.7%) rather than daily (1.3%; 1.3%).

**Table 2:** Frequency and quantity of past 12-month cannabis use according to cannabis type

	Cannabis type			
	High potency/indoor grown (*n* = 190)	Low potency/outdoor grown (*n* = 108)	Hash/resin (*n* = 21)	Cannabis oil/concentrates (*n* = 17)
Frequency				
Less than monthly (*n* = 149)	62 (41.6%)	61 (40.9%)	13 (8.7%)	13 (8.7%)
At least monthly (*n* = 111)	65 (58.6%)	36 (32.4%)	7 (6.3%)	3 (2.7%)
Daily (*n* = 76)	63 (82.9%)	11 (14.5%)	1 (1.3%)	1 (1.3%)
Quantity (joints/cones on typical occasion)				
≤ 1 (*n* = 73)	40 (54.8%)	22 (30.1%)	7 (9.6%)	4 (5.5%)
2–5 (*n* = 147)	86 (58.5%)	42 (28.6%)	10 (6.8%)	9 (6.1%)
5 > (*n* = 116)	64 (55.2%)	44 (37.9%)	4 (3.4%)	4 (3.4%)

In terms of quantity of use, a similar percentage of those who used ≤ 1 (54.8%), 2–5 (58.5%), and > 5 (55.2%) joints/cones of cannabis involved high-potency/indoor-grown cannabis (Table 2). Meanwhile, there was a slightly larger proportion of those using > 5 joints/cones (37.9%) of low potency/indoor-grown cannabis than those using ≤ 1 joints/cones (30.1%) of low potency/indoor cannabis. The proportion of those using hash/resin and cannabis oil/concentrates either remained stable or reduced with increasing quantity.

### The Relationship Between Baseline Covariates and Use of Varying Types of Cannabis

Baseline characteristics of the cohort according to the primary type of cannabis used in the past 12 months are presented in Supplementary table 2. A series of univariable regressions were conducted to investigate the relationship between baseline covariates and the use of high-potency cannabis as the primary type of cannabis used in the past 12 months (Table 3). In terms of demographics, age at baseline was significantly associated with lower odds of primarily using high-potency cannabis over the past year (OR = 0.92, 95% CI = 0.88, 0.97, *p* < 0.01). Meanwhile, reporting cannabis use (OR = 2.56, 95% CI = 1.19, 5.51, *p* = 0.02), and daily cannabis use at baseline (OR = 2.97, 95% CI = 1.41, 6.23, *p* < 0.01) was associated with greater odds of using high-potency cannabis as their main type of cannabis in the past 12 months.

**Table 3:** Univariable regression analysis between high-potency cannabis as the most common type used in the past 12 months and baseline outcomes

Baseline measures	B	SE	Wald	df	*P*-value	OR	95% CI
Demographics							
Age*	− 0.08	0.03	9.41	1	< 0.01	0.92	0.88–0.97
Male	− 0.02	0.40	0.00	1	0.95	0.98	0.45–2.12
Born in Australia	0.23	0.43	0.29	1	0.59	1.26	0.54–2.91
Wage, salary, or business as a main source of income	− 0.69	0.46	2.20	1	0.14	0.50	0.20–1.25
Own or renting accommodation	0.12	0.37	0.10	1	0.75	1.13	0.54–2.34
Ever been in prison	0.29	0.37	0.62	1	0.43	1.34	0.65–2.75
Crime in the past month	0.36	0.37	0.97	1	0.32	1.43	0.70–2.94
Treatment at study admission							
Residential rehabilitation	0.34	0.52	0.43	1	0.51	1.41	0.51–3.90
Maintenance therapy	0.39	0.39	0.97	1	0.33	1.47	0.68–3.18
Detoxification	− 0.54	0.38	2.05	1	0.15	0.58	0.28–1.22
No treatment	− 0.08	0.49	0.03	1	0.87	0.92	0.35–2.42
Past month cannabis use							
Cannabis*	0.94	0.39	5.82	1	0.02	2.56	1.19–5.51
Daily cannabis use*	1.09	0.38	8.25	1	< 0.01	2.97	1.41–6.23
Past month other drug use							
Heroin	0.45	1.17	0.15	1	0.70	1.57	0.16–15.48
Amphetamines	− 0.08	0.39	0.04	1	0.85	0.93	0.43–1.99
Cocaine	− 0.19	0.36	0.27	1	0.60	0.83	0.41–1.69
Benzodiazepines	0.45	0.37	1.51	1	0.22	1.57	0.77–3.24
Hallucinogens	0.72	0.77	0.87	1	0.35	2.05	0.45–9.23
Inhalants	− 0.15	1.13	0.02	1	0.89	0.86	0.09–7.90
Other opiates	0.72	0.43	2.76	1	0.10	2.04	0.88–4.74
Alcohol	− 0.16	0.37	1.18	1	0.67	0.86	0.42–1.75
Tobacco	− 19.71	15,191.51	0.00	1	0.99	0.00	0.00–0.00
Drug related harms							
Past month injection related problems	− 0.22	0.44	0.26	1	0.61	0.80	0.34–1.89
Ever overdose	0.29	0.36	0.63	1	0.43	0.66	0.66–2.72
Past 12-month overdose	0.56	0.45	1.55	1	0.21	0.72	0.72–4.26
Mental and physical health							
Mental Component Summary (MCS) score	0.47	0.37	1.62	1	0.21	1.61	0.78–3.33
Physical Component Summary (PCS) score	0.15	0.66	0.05	1	0.82	1.16	0.32–4.21
Past month major depression	0.33	0.46	0.51	1	0.48	1.39	0.57–3.38
Lifetime post-traumatic stress disorder (PTSD)	− 0.05	0.37	0.01	1	0.91	0.96	0.47–1.98
Antisocial personality disorder (ASPD)	0.11	0.44	0.07	1	0.80	1.12	0.47–2.67
Borderline personality disorder (BPD)	0.57	0.38	2.26	1	0.13	1.76	0.84–3.69
Suicide history							
Past month suicidal ideation	0.63	0.57	1.24	1	0.27	1.89	0.62–5.75
Ever attempt	0.17	0.39	0.19	1	0.66	1.19	0.56–2.52

***Statistically significant at 0.05

Due to low cell count, it was not possible to determine associations between past month use of antidepressants and high-potency cannabis as the primary type used in the past 12 months

The multivariable model was determined to be statistically significant when compared to the null model *χ*^2^(3) = 18.83, *p* < 0.01, where 14.2% of variation in cannabis type was explained by the model. After accounting for all significant variables at baseline, only age at baseline (OR = 0.92, 95% CI = 0.87, 0.97, *p* < 0.01) was associated with lower odds of high-potency cannabis being used as the primary type of cannabis in the past 12 months (Table 4).

**Table 4:** Multivariable logistical regression model for associations between baseline outcomes and high-potency cannabis as the most common type used in the past 12 months

Baseline measures	B	SE	Wald	df	*P*-value	OR	95% CI
Age*	− 0.08	0.03	9.31	1	< 0.01	0.92	0.87–0.97
Past month cannabis use	0.66	0.51	1.67	1	0.20	1.94	0.71–5.31
Past month daily cannabis use	0.68	0.48	1.98	1	0.16	1.98	0.77–5.10
Constant	1.53	0.35	19.28	1	< 0.01		

*Statistically significant where *p*-value is less than 0.05/3 = 0.02

There were no significant three- or two-way interactions between age, past month cannabis use, and past month daily cannabis use

### The Relationship Between 18–20-Year Covariates and Varying Cannabis Products

Eighteen- to twenty-year characteristics of the cohort according to the primary type of cannabis used in the past 12 months are presented in Supplementary table 3. A series of univariable regressions were conducted to investigate the relationship between 18–20-year variables and high-potency cannabis being used as the primary type of cannabis in the past 12 months (Table 5). In terms of demographics, owning or renting accommodation was associated with lower odds of high-potency cannabis being used as the primary type of cannabis in the past 12 months (OR = 0.34, 95% CI = 0.16, 0.70, *p* < 0.01). In terms of criminal involvement, prison since last interview was associated with greater odds of high-potency cannabis as the primary type of cannabis in the past year (OR = 3.43, 95% CI = 1.27, 9.26, *p* < 0.01). Amphetamine use (OR = 2.99, 95% CI = 1.18, 7.55, *p* = 0.02), and past 12-month PTSD diagnosis (OR = 4.63, 95% CI = 1.06, 20.18, *p* = 0.04) was also associated with greater odds of high-potency cannabis as the primary type of cannabis in the past year.

**Table 5:** Univariable regression analysis between high-potency cannabis as the most common type used in the past 12 months and 18–20-year outcomes

18–20-year measures	*B*	SE	Wald	df	*P*-value	OR	95% CI
Demographics							
Wage, salary, or business as main source of income	− 0.74	0.41	3.17	1	0.08	0.48	0.21–1.09
Own or renting accommodation*	− 1.09	0.37	8.51	1	< 0.01	0.34	0.16–0.70
Prison since last interview*	1.23	0.51	5.91	1	0.02	3.43	1.27–9.26
Crime in the past month	0.38	0.52	0.54	1	0.46	1.46	0.53–4.05
Past month cannabis use							
Cannabis	0.68	0.39	3.03	1	0.08	1.98	0.92–4.25
Daily cannabis use	0.68	0.38	3.22	1	0.07	1.99	0.94–4.21
Past month other drug use							
Heroin	0.30	0.42	0.51	1	0.47	1.35	0.60–3.06
Amphetamines*	1.09	0.47	5.34	1	0.02	2.99	1.18–7.55
Cocaine	− 0.73	0.72	1.05	1	0.31	0.48	0.12–1.95
Benzodiazepines	0.38	0.38	0.99	1	0.32	1.47	0.69–3.11
Antidepressants	1.10	0.56	3.92	1	0.05	3.01	1.10–8.97
Other opiates	0.75	0.45	2.80	1	0.09	2.12	0.88–5.13
Alcohol	− 0.07	0.36	0.04	1	0.85	0.93	0.46–1.90
Gabapentin	− 1.56	1.43	1.19	1	0.28	0.21	0.01–3.45
Tobacco	− 0.39	0.57	0.46	1	0.50	0.68	0.22–2.08
Drug-related harms							
Injection-related problems	− 0.39	0.57	0.46	1	0.50	0.68	0.22–2.08
Ever overdose	0.27	0.40	0.45	1	0.50	1.31	0.60–2.87
Drug dependence							
Cannabis dependence	0.38	0.48	0.63	1	0.43	1.47	0.57–3.77
Heroin dependence	0.60	0.57	1.11	1	0.29	1.81	0.60–5.49
Mental and physical health							
Mental Component Summary (MCS) score (< 30)	0.41	0.48	0.73	1	0.39	1.51	0.59–3.89
Physical Component Summary (PCS) score (< 30)	0.48	0.65	0.56	1	0.46	1.62	0.46–5.74
Past month major depression	0.52	0.57	0.82	1	0.36	1.67	0.55–5.09
Past 12-month PTSD*	1.53	0.75	4.15	1	0.04	4.63	1.06–20.18
Suicide history							
Past month suicidal ideation	0.39	0.84	0.21	1	0.64	1.47	0.29–7.58
Ever attempt	0.67	0.37	3.37	1	0.07	1.96	0.96–4.02
Cognition score	–0.21	0.15	1.86	1	0.17	0.81	0.60–1.10

***Statistically significant at 0.05

Due to low cell count, it was not possible to determine associations between past month use of lyrica, hallucinogens, inhalants, NEPS, as well as past 12-month overdose and high-potency cannabis as the primary type used in the past 12 months

The multivariable model was determined to be statistically significant when compared to the null model *χ*^2^(7) = 37.71, *p* < 0.01, where 28.1% of variation in cannabis type was explained by the model. After accounting for all significant variables at 18–20 years and baseline, 18–20-year covariates were no longer significantly associated with high-potency cannabis being used as the primary type of cannabis in the past 12 months (Table 6). Thus primary use of high-potency cannabis was not associated with more severe mental health, substance use, or cognitive outcomes.

**Table 6:** Multivariable logistical regression model for associations between 18–20-year outcomes and high-potency cannabis as the most common type used in the past 12 months

18–20-year measures^a^	*B*	SE	Wald	df	*P*–value	OR	95% CI
Privately own or renting accommodation	–1.10	0.45	5.75	1	0.02	0.34	0.14–0.82
Prison since last interview	0.68	0.58	1.39	1	0.24	1.98	0.64–6.17
Past month amphetamine use	0.73	0.53	1.89	1	0.17	2.08	0.73–5.91
PTSD	1.41	0.80	3.11	1	0.08	4.08	0.86–19.44
Constant	3.01	1.09	7.66	1	0.01		

^a^Controlling for significant baseline measures included in Table 22

^*^Statistically significant where *p*-value is less than 0.05/4 = 0.01

## Discussion

Considering that cannabis use is common among those with OUD, this was one of the first studies to assess the prevalence of, and long-term associations with, the use of varying cannabis products among a cohort of people with heroin dependence. Cannabis use was common, where at 18–20 years post-baseline, 397 (99.0%) had ever used cannabis, and 209 (52.1%) had used in the past 12 months. High-potency/indoor-grown cannabis was most used since first time use and over the past 12 months, with fewer commonly using low potency/outdoor grown, and very few using other types of cannabis. Univariable regression revealed that baseline measures such as age, past month cannabis use, and past month daily cannabis use were associated with high-potency cannabis being used as the primary form of cannabis in the past 12 months. There was also some evidence for a relationship with 18–20-year covariates, including owning or renting accommodation, being in prison since last interview, past month amphetamine use, and a PTSD diagnosis. However, the inclusion of these factors in a multivariable model determined age at baseline to be the only significant associate with common use of high-potency cannabis.

The high prevalence of high-potency/indoor-grown cannabis commonly used by the cohort is largely consistent with other forms of data. Most recent findings from an annual illicit drug monitoring system that regularly interviews people who inject drugs within Australia showed that 91% of those reporting recent cannabis use were consuming indoor grown, 37% outdoor grown, and only up to 6% using hash products (Sutherland et al., 2021). Although international data suggests that there is a greater number of countries that solely produce outdoor-grown products, regions with indoor cultivations such as Europe, North America, and Australia have experienced a marked increase in indoor products over the past 10 years (World Drug Report, 2021), potentially explaining the overall increase in THC concentrations within herbal cannabis worldwide (Freeman et al., 2021). Furthermore, while there has been an increase in cannabis resin seized within Europe and Middle East/South-West Asia over the past 40 years, prevalence of seized resin samples has remained relatively low elsewhere (World Drug Report, 2021). As for concentrates, low prevalence may be explained by limited availability within Australia compared to regions such as the USA and Canada (Goodman et al., 2020), or lower consumer demand among older adults (Ueno et al., 2021).

While just as many of the cohort were using low potency/outdoor grown less than monthly, over 80% of those using cannabis daily reported use of high potency/indoor grown. In support, it has been demonstrated that THC exhibits dose-related effects on drug reinforcement (Curran et al., 2016) and that there appears to be a strong positive association between THC in cannabis markets and first-time cannabis admissions to drug treatment centres (Freeman et al., 2018). This effect may be more salient in this population given preclinical findings suggesting that the use of opioid agonists increases the reward induced by THC (Wiese & Wilson-Poe, 2018). Furthermore, when combining these categories (high potency/indoor grown, hash/resin, and cannabis oil/concentrates) together, those reporting past month daily cannabis use or cannabis dependence were no more likely to report having used high-potency cannabis as their main type of cannabis after accounting for other variables. Overall, these findings offer partial support for the argument that high-potency cannabis use is associated with more persistent or severe cannabis use.

The current finding of an association between high-potency cannabis use and age at baseline is largely consistent with prior evidence (Hines et al., 2020; Korf et al., 2007). A possible explanation for this is that young people appear to be at the greatest risk of developing cannabis dependence when they are using high-potency cannabis (Freeman & Winstock, 2015), which may reinforce further use of more potent cannabis products. Alternatively, high-potency cannabis products were less accessible during a time when older participants were initiating cannabis, and older people therefore may have been more likely to initiate and/or continue using lower-potency products compared to younger people. Nevertheless, these findings should inform intervention programs that aim to reduce the use of more potent products among young people, thus reducing associated harms (Fischer et al., 2017).

In contrast to prior research, there was no evidence of an association between high-potency cannabis use with poorer cognitive performance (Curran et al., 2002), the use of other drugs (Hines et al., 2020), and major depression (Chan et al., 2017). These inconsistencies could be explained by differences in study populations and a possible ceiling effect, whereby people with OUD are already experiencing adverse health outcomes (e.g. poorer mental health, cognitive impairment) to a high degree, and may therefore be less likely to exhibit differences according to use of cannabis products. Alternatively, there may have been insufficient power to detect differences in some health outcomes according to cannabis type. For instance, only 20 participants reporting past 12-month cannabis use experienced recent suicidal ideation. Nevertheless, this highlights the need to explore the dose dependent effects of THC beyond populations of relatively healthy adults.

There are a few factors that must be taken into consideration when interpreting the current findings. Firstly, while the measures of using different cannabis products may be a proxy for exposure to different doses of THC and CBD, the study was unable to determine precise measures of cannabinoid content. While the literature supports the combination of high-potency/indoor-grown herbal, cannabis oil/concentrates, and hash/resin into a high-potency category, common use of the latter types was rare. As a result, the findings of a relationship between covariates and high-potency cannabis use may reflect the use of high-potency/indoor-grown herbal specifically. Also, while hash/resin are currently considered to be high-potency products following an observed increase in THC over the past few decades, common use may have been during a time when they were comparable to low-potency/indoor-grown products (Freeman et al., 2021). These points contribute to the broader limitations of accurately measuring THC content within observational studies which are made further difficult by existing illegal cannabis markets. Measures of THC exposure can be further improved by gas chromatography analysis of seized samples, or the implementation of the 5 mg standard THC unit like that used with alcohol (Freeman & Lorenzetti, 2021). However, unlike alcohol, there is no valid biomarker of exposure.

Despite these limitations, this is the first study to have assessed the use of various cannabis products among people with heroin dependence. Cannabis use is common among this population, and although there has been emerging interest in cannabis as a substitute for opioids (Humphreys & Saitz, 2019), there is little understanding of the exposure to high-potency cannabis products and potential harms. We did not find evidence to suggest a beneficial effect of low-potency cannabis use on physical or mental health in this cohort, nor was there any suggestion of greater harm among those using high-potency cannabis. However, given the high prevalence of cannabis use among this population, there is a clear need for further long-term studies to monitor the effects of different cannabis products.

## Conclusions

In conclusion, high potency/indoor grown was by far the most common type of cannabis used among a cohort of people with a history of heroin dependence. Those primarily using high-potency cannabis relative to low-potency cannabis were more likely to have been younger, but cannabis type was not associated with cognitive performance, mental health diagnosis, or the use of other substances. Nevertheless, clinicians should monitor high THC consumption given the increased risk of harms among an already vulnerable population.

## Supplementary Information

Below is the link to the electronic supplementary material.

Supplementary file1 (DOCX 30 KB)

## Data Availability

Data are currently unavailable for this study.

## References

[CR1] Bhattacharyya S, Morrison PD, Fusar-Poli P, Martin-Santos R, Borgwardt S, Winton-Brown T, Nosarti C, O' Carroll CM, Seal M, Allen P, Mehta MA, Stone JM, Tunstall N, Giampietro V,  Kapur S, Murray RM, Zuardi AW, Crippa JA, Atakan Z, McGuire PK (2010). Opposite effects of Δ-9-tetrahydrocannabinol and cannabidiol on human brainfunction and psychopathology. Neuropsychopharmacology.

[CR2] Bonaccorso S, Ricciardi A, Zangani C, Chiappini S, Schifano F (2019). Cannabidiol (CBD) use in psychiatric disorders: A systematic review. Neurotoxicology.

[CR3] Campos AC, Moreira FA, Gomes FV, Del Bel EA, Guimarães FS (2012). Multiple mechanisms involved in the large-spectrum therapeutic potential of cannabidiol in psychiatric disorders. Philosophical Transactions of the Royal Society B: Biological Sciences.

[CR4] Chan GCK, Hall W, Freeman TP, Ferris J, Kelly AB, Winstock A (2017). User characteristics and effect profile of butane hash oil: An extremely high-potency cannabis concentrate. Drug and Alcohol Dependence.

[CR5] Compton WM, Valentino RJ, DuPont RL (2021). Polysubstance use in the U.S. opioid crisis. Mol Psychiatry.

[CR6] Craft S, Winstock A, Ferris J, Mackie C, Lynskey MT, Freeman TP (2019). Characterising heterogeneity in the use of different cannabis products: Latent class analysis with 55 000 people who use cannabis and associations with severity of cannabis dependence. Psychological medicine.

[CR7] Curran HV, Brignell C, Fletcher S, Middleton P, Henry J (2002). Cognitive and subjective dose-response effects of acute oral Delta 9-tetrahydrocannabinol (THC) in infrequent cannabis users. Psychopharmacology (berl).

[CR8] Curran HV, Freeman TP, Mokrysz C, Lewis DA, Morgan CJA, Parsons LH (2016). Keep off the grass? Cannabis, cognition and addiction. Nature Reviews Neuroscience.

[CR9] D'Souza DC, Perry E, MacDougall L, Ammerman Y, Cooper T, Wu YT, Braley G, Gueorguieva R, Krystal JH (2004). The psychotomimetic effects of intravenous delta-9-tetrahydrocannabinol in healthy individuals: Implications for psychosis. Neuropsychopharmacology.

[CR10] Darke S, Hall W, Wodak A, Heather N, Ward J (1992). Development and validation of a multi-dimensional instrument for assessing outcome of treatment among opiate users: The Opiate Treatment Index. British Journal of Addiction.

[CR11] Darke S, Ross J, Hall W (1996). Overdose among heroin users in Sydney, Australia: I. Prevalence and correlates of non-fatal overdose. Addiction.

[CR12] Degenhardt L, Charlson F, Ferrari A, Santomauro D, Erskine H, Mantilla-Herrara A, Whiteford H, Leung J, Naghavi M, Griswold M, Rehm J, Hall W, Sartorius B, Scott J, Vollset S, Knudsen AK, Haro JM, Patton G, Kopec J, Carvalho Malta D, Topor-Madry R, McGrath G, Haagsma J, Allebeck P, Phillips M, Salomon J, Hay S, Foreman K, Lim S, Mokdad A, Smith M, Gakidou E, Murray C, Vos T (2018). The global burden of disease attributable to alcohol and drug use in 195 countries and territories, 1990–2016: a systematic analysis for the Global Burden of Disease Study 2016. The Lancet Psychiatry.

[CR13] Demirakca T, Sartorius A, Ende G, Meyer N, Welzel H, Skopp G, Mann K, Hermann D (2011). Diminished gray matter in the hippocampus of cannabis users: Possible protective effects of cannabidiol. Drug and Alcohol Dependence.

[CR14] Di Forti M, Quattrone D, Freeman TP, Tripoli G, Gayer-Anderson C, Quigley H, Rodriguez V, Jongsma HE, Ferraro L, La Cascia C, La Barbera D, Tarricone I, Berardi D, Szöke A, Arango C, Tortelli A, Velthorst E, Bernardo M, Del-Ben CM, Menezes PR, Selten J-P, Jones PB, Kirkbride JB, Rutten BPF, de Haan L, Sham PC, van Os J, Lewis CM, Lynskey M, Morgan C, Murray RM, Amoretti S, Arrojo M, Baudin G, Beards S, Bernardo M, Bobes J, Bonetto C, Cabrera B, Carracedo A, Charpeaud T, Costas J, Cristofalo D, Cuadrado P, Díaz-Caneja CM, Ferchiou A, Franke N, Frijda F, García Bernardo E, Garcia-Portilla P, González E, Hubbard K, Jamain S, Jiménez-López E, Leboyer M, López Montoya G, Lorente-Rovira E, Marcelino Loureiro C, Marrazzo G, Martínez C, Matteis M, Messchaart E, Moltó MD, Nacher J, Olmeda MS, Parellada M, González Peñas J, Pignon B, Rapado M, Richard J-R, Rodríguez Solano JJ, Roldán Díaz L, Ruggeri M, Sáiz PA, Sánchez E, Sanjuán J, Sartorio C, Schürhoff F, Seminerio F, Shuhama R, Sideli L, Stilo SA, Termorshuizen F, Tosato S, Tronche A-M, van Dam D, van der Ven E (2019). The contribution of cannabis use to variation in the incidence of psychotic disorder across Europe (EU-GEI): a multicentre case-control study. The Lancet Psychiatry.

[CR15] ElSohly MA, Mehmedic Z, Foster S, Gon C, Chandra S, Church JC (2016). Changes in cannabis potency over the last 2 decades (1995–2014): Analysis of current data in the United States. Biological Psychiatry.

[CR16] Englund A, Morrison PD, Nottage J,  Hague D, Kane F, Bonaccorso S, Stone JM, Reichenberg A,  Brenneisen R, Holt D, Feilding A, Walker L, Murray RM, Kapur S (2013). Cannabidiol inhibits THC-elicited paranoid symptoms and hippocampal-dependent memory impairment. J Psychopharmacol.

[CR17] Englund A, Oliver D, Chesney E, Chester L, Wilson J, Sovi S, De Micheli A, Hodsoll J, Fusar-Poli P, Strang J, Murray RM, Freeman TP, McGuire P (2022). Does cannabidiol make cannabis safer? A randomised, double-blind, cross-over trial of cannabis with four different CBD:THC ratios. Neuropsychopharmacol..

[CR18] Fischer B, Russell C, Sabion P, van den Brink W, Le Foll B, Hall W, Rehm J, Room R (2017). Lower-risk cannabis use guidelines: A comprehensive update of evidence and recommendations. Am J Public Health.

[CR19] Freeman A, Petrilli K, Lees R, Hindocha C, Mokrysz C, Curran HV, Saunders R, Freeman TP (2019). How does cannabidiol (CBD) influence the acute effects of delta-9-tetrahydrocannabinol (THC) in humans? A systematic review. Neurosci Biobehav Rev.

[CR20] Freeman TP, Hindocha C, Green SF, Bloomfield MAP (2019). Medicinal use of cannabis based products and cannabinoids. BMJ.

[CR21] Freeman TP, Craft S, Wilson J, Stylianou S, ElSohly M, Di Forti M, Lynskey MT (2021). Changes in delta-9-tetrahydrocannabinol (THC) and cannabidiol (CBD) concentrations in cannabis over time: Systematic review and meta-analysis. Addiction.

[CR22] Freeman TP, Hindocha C, Baio G, Shaban NDC, Thomas EM, Astbury D, Freeman AM, Lees R, Craft S, Morrison PD, Bloomfield MAP, O'Ryan D, Kinghorn J, Morgan CJA, Mofeez A, Curran HV (2020). Cannabidiol for the treatment of cannabis use disorder: A phase 2a, double-blind, placebo-controlled, randomised, adaptive Bayesian trial. The Lancet Psychiatry.

[CR23] Freeman TP, Lorenzetti V (2021). A standard THC unit for reporting of health research on cannabis and cannabinoids. The Lancet. Psychiatry.

[CR24] Freeman TP, Morgan CJ, Hindocha C, Schafer G, Das RK, Curran HV (2014). Just say 'know': How do cannabinoid concentrations influence users’ estimates of cannabis potency and the amount they roll in joints?. Addiction.

[CR25] Freeman TP, Van Der Pol P, Kuijpers W, Wisselink J, Das RK, Rigter S, Laar MV, Griffiths P, Swift W, Niesink R, Lynskey MT (2018). Changes in cannabis potency and first-time admissions to drug treatment: A 16-year study in the Netherlands. Psychological Medicine.

[CR26] Freeman TP, Winstock AR (2015). Examining the profile of high-potency cannabis and its association with severity of cannabis dependence. Psychological Medicine.

[CR27] Goodman S, Wadsworth E, Leos-Toro C, Hammond D (2020). Prevalence and forms of cannabis use in legal vs. illegal recreational cannabis markets. International Journal of Drug Policy.

[CR28] Hanuš LO, Meyer SM, Muñoz E, Taglialatela-Scafati O, Appendino G (2016). Phytocannabinoids: A unified critical inventory. Natural Products Reports.

[CR29] Hasin D, Walsh C (2020). Cannabis use, cannabis use disorder, and comorbid psychiatric illness: A narrative review. Journal of Clinical Medicine.

[CR30] Hines LA, Freeman TP, Gage SH, Zammit S, Hickman M, Cannon M, Munafo M, MacLeod J, Heron J (2020). Association of high-potency cannabis use with mental health and substance use in adolescence. JAMA Psychiatry.

[CR31] Hsieh S, McGrory S, Leslie F, Dawson K, Ahmed S, Butler CR,  Rowe JB, Mioshi E, Hodges JR (2015). The Mini-Addenbrooke’s Cognitive Examination: A new assessment tool for dementia. Dement Geriatr Cogn Disord.

[CR32] Humphreys K, Saitz R (2019). Should physicians recommend replacing opioids with cannabis?. JAMA.

[CR33] Hurd YL, Spriggs S, Alishayev J, Winkel G, Gurgov K, Kudrich C, Oprescu AM, Salsitz E (2019). Cannabidiol for the reduction of cue-induced craving and anxiety in drug-abstinent individuals with heroin use disorder: A double-blind randomized placebo-controlled trial. American Journal of Psychiatry.

[CR34] Korf DJ, Benschop A, Wouters M (2007). Differential responses to cannabis potency: A typology of users based on self-reported consumption behaviour. International Journal of Drug Policy.

[CR35] Kral AH, Wenger L, Novak SP, Chu D, Corsi KF, Coffa D, Shapiro B, Bluthenthal RN (2015). Is cannabis use associated with less opioid use among people who inject drugs?. Drug & Alcohol Dependence.

[CR36] Leung J, Stjepanović D, Dawson D, Hall WD (2021). Do cannabis users reduce their THC dosages when using more potent cannabis products?. A review. Front Psychiatry.

[CR37] Loranger AW, Janca A, Sartorius N (1997). Assessment and diagnosis of personality disorders: The ICD-10 international personality disorder examination (IPDE).

[CR38] Mackie CJ, Wilson J, Freeman TP, Craft S, Escamilla De La Torre T, Lynskey MT (2021). A latent class analysis of cannabis use products in a general population sample of adolescents and their association with paranoia, hallucinations, cognitive disorganisation and grandiosity. Addictive Behaviors.

[CR39] Marel, C., Wilson, J., Darke, S., Ross, J., Slade, T., Haber, P. S., Haasnoot, K., Visontay, R., Keaveny, M., Tremonti, C., Mills, K. L., & Teesson, M. (2023). Patterns and predictors of heroin use, remission, and psychiatric health among people with heroin dependence: Key findings from the 18–20-year follow-up of the Australian Treatment Outcome Study (ATOS). *International Journal of Mental Health and Addiction*. 10.1007/s11469-022-01006-6 10.1007/s11469-022-01006-6 PMC9847452 36688114

[CR40] McPartland JM, Duncan M, Di Marzo V, Pertwee RG (2015). Are cannabidiol and Delta(9) -tetrahydrocannabivarin negative modulators of the endocannabinoid system? A Systematic Review. British Journal of Pharmacology.

[CR41] Morgan CJA, Curran HV (2008). Effects of cannabidiol on schizophrenia-like symptoms in people who use cannabis. British Journal of Psychiatry.

[CR42] Morgan CJA, Freeman TP, Schafer GL, Curran HV (2010). Cannabidiol attenuates the appetitive effects of Δ9-Tetrahydrocannabinol in humans smoking their chosen cannabis. Neuropsychopharmacology.

[CR43] Morgan CJA, Gardener C, Schafer G, Swan S, Demarchi C, Freeman TP, Warrington P, Rupasinghe I, Ramoutar A, Tan N, Wingham G, Lewis S, Curran HV (2012). Sub-chronic impact of cannabinoids in street cannabis on cognition, psychotic-like symptoms and psychological well-being. Psychol Med.

[CR44] Morgan CJA, Schafer G, Freeman TP, Curran HV (2010). Impact of cannabidiol on the acute memory and psychotomimetic effects of smoked cannabis: Naturalistic study: Naturalistic study [corrected]. British Journal of Psychiatry.

[CR45] O'Brien R (2007). A Caution Regarding Rules of Thumb for Variance Inflation Factors. Quality & Quantity.

[CR46] Peacock, A., Uporova, J., Karlsson, T., Gibbs, D., Swanton, R., Kelly, G., Price, O., Bruno, R., Dietze, P., Lenton, S., Salom, C., Degenhardt, L., & Farrell, M. (2019). *Australian Drug Trends 2019: Key findings from the National Illicit Drug Reporting System (IDRS) Interviews*. Retrieved from Sydney: http://handle.unsw.edu.au/1959.4/unsworks_61766

[CR47] Potter DJ (2013). A review of the cultivation and processing of cannabis (Cannabis sativa L.) for production of prescription medicines in the UK. Drug Testing and Analysis.

[CR48] Potter DJ, Hammond K, Tuffnell S, Walker C, Di Forti M (2018). Potency of Δ9–tetrahydrocannabinol and other cannabinoids in cannabis in England in 2016: Implications for public health and pharmacology. Drug Testing and Analysis.

[CR49] Raber JC, Elzinga S, Kaplan C (2015). Understanding dabs: Contamination concerns of cannabis concentrates and cannabinoid transfer during the act of dabbing. The Journal of Toxicological Sciences.

[CR50] Rosic T, Kapoor R, Panesar B, Naji L, Chai DB,  Sanger N, Marsh DC, Worster A, Thabane L, Samaan Z (2021). The association between cannabis use and outcome in pharmacological treatment for opioid use disorder. Harm Reduct J.

[CR51] Ross, J., Teesson, M., Darke, S., Lynskey, M., Hetherington, K., Mills, K., Williamson, A., & Fairbairn, S. (2002). Characteristics of heroin users entering three treatment modalities in New South Wales: Baseline findings from the Australian Treatment Outcome Study (ATOS). *National Drug Alcohol Research Centre Technical Report No, 139*.

[CR52] Sutherland, R., Uporova, J., Chandrasena, U., Price, O., Karlsson, A., Gibbs, D., Swanton, R., Bruno, R., Dietze, P., Lenton, S., Salom, C., Daly, C., Thomas, N., Juckel, J., Agramunt, S., Wilson, Y., Woods, E., Moon, C., Degenhardt, L., Farrell, M., & Peacock, A. (2021). *Australian Drug *Trends* 2021: Key Findings from the National Illicit Drug Reporting System (IDRS) Interviews.* Retrieved from

[CR53] Ueno LF, Mian MN, Altman BR, Giandelone E, Luce M, Earleywine M (2021). Age-related differences in cannabis product use. Journal of Psychoactive Drugs.

[CR54] Ware JE, Kosinski M, Keller SD (1996). A 12-item short-form health survey: Construction of scales and preliminary tests of reliability and validity. Medical Care.

[CR55] Wiese B, Wilson-Poe AR (2018). Emerging evidence for cannabis’ role in opioid use disorder. Cannabis and Cannabinoid Research.

[CR56] World Drug Report. (2021). (United Nations publication, Sales No. E.21.XI.8).

[CR57] World Health Organisation. (1997). *Composite International Diagnostic Interview (CIDI) Core Version 2.1, 12 month version*. Retrieved from Geneva: World Health Organisation:

